# Big Things, Small Packages: An Update on Microalgae as Sustainable Sources of Nutraceutical Peptides for Promoting Cardiovascular Health

**DOI:** 10.1002/gch2.202200162

**Published:** 2023-01-31

**Authors:** Chukwunonso E. C. C. Ejike, Timothy P. C. Ezeorba, Obinna Ajah, Chibuike C. Udenigwe

**Affiliations:** ^1^ Department of Medical Biochemistry Faculty of Basic Medical Sciences Alex Ekwueme Federal University Ndufu‐Alike Ebonyi State 482131 Nigeria; ^2^ Department of Biochemistry Faculty of Biological Sciences University of Nigeria Nsukka Enugu State 410001 Nigeria; ^3^ Department of Biochemistry College of Natural Sciences Michael Okpara University of Agriculture Umudike Abia State 440101 Nigeria; ^4^ School of Nutrition Sciences Faculty of Health Sciences University of Ottawa Ottawa Ontario K1H 8M5 Canada; ^5^ Department of Chemistry and Biomolecular Sciences Faculty of Science University of Ottawa Ottawa Ontario K1N 6N5 Canada

**Keywords:** bioactive peptides, cardiovascular health, microalgae, nutraceuticals, sustainable proteins

## Abstract

In 2017, a review of microalgae protein‐derived bioactive peptides relevant in cardiovascular disease (CVD) management was published. Given the rapid evolution of the field, an update is needed to illumininate recent developments and proffer future suggestions. In this review, the scientific literature (2018–2022) is mined for that purpose and the relevant properties of the identified peptides related to CVD are discussed. The challenges and prospects for microalgae peptides are similarly discussed. Since 2018, several publications have independently confirmed the potential to produce microalgae protein‐derived nutraceutical peptides. Peptides that reduce hypertension (by inhibiting angiotensin converting enzyme and endothelial nitric oxide synthase), modulate dyslipidemia and have antioxidant and anti‐inflammatory properties have been reported, and characterized. Taken together, future research and development investments in nutraceutical peptides from microalgae proteins need to focus on the challenges of large‐scale biomass production, improvement in techniques for protein extraction, peptide release and processing, and the need for clinical trials to validate the claimed health benefits as well as formulation of various consumer products with the novel bioactive ingredients.

## Graphical abstract

Microalgae biomass has tremendous potential as a sustainable source of alternative proteins. Enzymatic processing of microalgae proteins yields structurally diverse peptides with multiple biofunctional properties. This review discusses recent developments in microalgae protein‐derived bioactive peptides relevant in cardiovascular disease management, and the challenges to practical application and areas that need additional research and development.

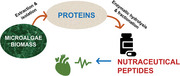

## 1 Introduction

Cardiovascular diseases (CVDs) are a group of etiologically intertwined disorders that affect the heart and blood vessels. The World Health Organization (WHO) reports that annually 17.9 million people die from CVDs, representing an estimated 32% of all deaths globally.^[^ ^1^ ^]^ The role of oxidative stress, dyslipidemia and hypertension as major risk factors for CVDs are established in the literature. Natural bioactives that have the capacity for positively modulating CVDs risk factors therefore have very significant roles in CVDs prevention and management. Peptides are short fragments of proteins made up of about 2–20 amino acid residues. Bioactive peptides are peptides with capacity to interfere with biochemical pathways in ways that improve health. Also known as nutraceutical peptides and cryptic peptides (cryptides), these molecules can be obtained through a variety of means, including normal digestion of food proteins in the gut, autolysis in food, chemical and enzymatic hydrolysis, microbial fermentation, and physical processing. Microalgae are currently known to be treasure chests of bioactive peptides.^[^ ^2^ ^]^

Given the high prevalence of cardiovascular diseases globally; the pivot towards the use of microalgae for a variety of nutrition and health purposes; and the emerging research into bioactive peptides, we reviewed the literature on bioactive peptides that have reported activity related to cardiovascular diseases prevention and management.^[^ ^2^ ^]^ The paper was well received and has been generously used and cited by researchers around the world. Owing to the rapid turnover of research on bioactive peptides from microalgae, it became important to update the paper we published earlier. Therefore, the objective of this review is to discuss recent scientific advances, opportunities, and challenges on the processing of microalgae protein to produce peptides with potential impact in the management of hypertension, oxidative stress, dyslipidemia, and inflammation.

Data included in this review were derived from a search of published literature on bioactive peptides with reported usefulness in managing or preventing CVDs. Major online databases, including PubMed/Medline, ScienceDirect, and Google Scholar, were searched for literature published between 2018 and 2022, in the English language. From the initial papers identified to be relevant to the review, a search of their reference lists yielded additional titles, which were then included in the list of papers assessed. A total of fifty‐four articles (original research, reviews, and book chapters) which met the inclusion criteria were identified. Of the 54 articles, 21 original research articles were selected and from them, data for the core of this review were extracted.

## 2 Microalgae Proteins as Sustainable Sources of BAPs

**Figure 1:**
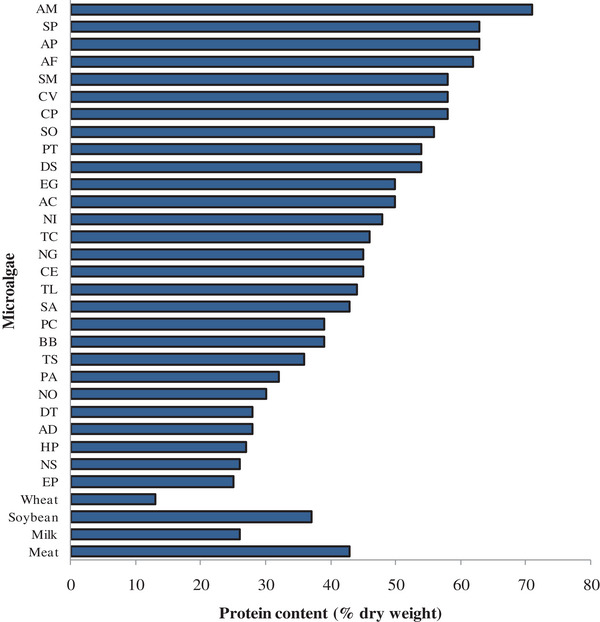
Comparison of the protein content of various microalgae species with those of commonnutritional protein sources. Abbreviations used: AC, Anabaena cylindrica; AD, *Acutodesmus dimorphus*; AF, *Aphanizomenon flos‐aquae*; AM, *Arthospira maxima*; AP, *Arthrospira platensis*; BB, *Botryococcus braunii*; CE, *Chlorella ellipsoidea;* CP, *Chlorella pyrenoidosa*; CV, *Chlorella vulgaris*; DS, *Dunaliella salina*; DT, *Dunaliella tertiolecta*; EG, *Euglena gracilis*; EP, *Entomoneis punctulata*; HP, *Haematococcus pluvialis*; NG, *Nannochloropsis gaditana*; NI, *Navicula incerta*; NO, *Neochloris oleoabundans*; NS, *Nannochloropsis sp*.; PA, *Porphyridium aerugineum*; PC, *Porphyridium cruentum*; PT, *Phaeodactylum tricornutum*; SA, *Scenedesmus almeriensis*; SM, *Spirulina maxima*; SO, *Scenedesmus obliquus*; SP, *Spirulina platensis*; SS, *Synechococcus sp*.; TC, *Tetraselmis chuii*; TL, *Tisochrysis lutea*; and TS, *Tetraselmis suecica*. Data were extracted from Ejike et al.;^[^ ^2^ ^]^ Li et al.;^[^ ^5^ ^]^ Acquah et al.;^[^ ^4^ ^]^ and the references therein.

Microalgae biomass has huge potentials as a source of food proteins. They are rich in proteins that are comparable to the proteins in conventional food proteins such as soybeans, eggs, and fish, in terms of quantity and quality.^[^ ^3^, ^4^ ^]^ For example, *Spirulina sp*. and *Chlorella sp*. are known to contain about 50–70% and 53–58% proteins, respectively depending on the strain.^[^ ^2^, ^5^ ^]^ Indeed, among the many species of algae, *Chlorella* and *Spirulina* species command the most demand in the global microalgae market. **Figure** **1** shows a comparison of the proteins of selected microalgae and those of traditional food sources rich in protein. Beyond the quantity of proteins available in microalgae, their high growth rate and protein turnover make them attractive. For instance, *Dunaliella sp*. can produce 50–100 times more protein per unit area than conventionally grown plants and animals that are protein sources.

**Figure 2:**
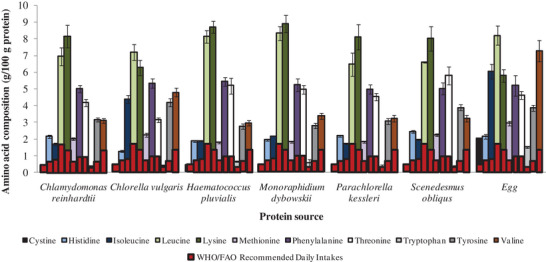
Amino acid composition of proteins in selected microalgae and chicken egg compared to the WHO/FAO recommended daily intakes (RDI) for a 70‐kg adult human for essential amino acids with RDI > 0.0. Data extracted from Siahbalaei et al.^[^ ^6^ ^]^

**Figure 3:**
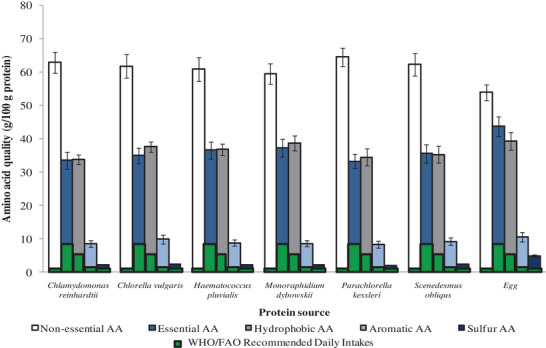
Relevant amino acid nutritional quality indicators for selected microalgae and chicken egg compared to WHO/FAO recommended daily intakes for a 70‐kg adult human. Data extracted from Siahbalaei et al.^[^ ^6^ ^]^

Li et al.^[^ ^5^ ^]^ reported that the amount of essential/indispensable amino acids of selected microalgae compared favorably with those of eggs (raw and cooked) and soybean (cooked) on the one hand, and the WHO/FAO reference on the other hand. Indeed, for histidine, isoleucine, leucine, lysine, threonine, and valine, *Nannochloropsis sp*., *Phaeodactylum tricornutum*, *Scenedesmus obliquus*, and *Arthrospira platensis* had more of the essential/indispensable amino acids compared to the reference values. Siahbalaei et al.^[^ ^6^ ^]^ further showed that microalgae proteins contain comparable amino acid profiles (including all 20 proteinogenic amino acids) and compositions to those of egg protein. Except for cystine and tryptophan, the amino acid composition per 100 g of microalgae protein provide much more amino acids than the FAO/WHO recommended daily intakes (RDI) (**Figure** **2**). The authors also found that for the major groupings of amino acids—essential amino acids, non‐essential amino acids, hydrophobic amino acids, aromatic amino acids, sulfur containing amino acids, etc.—microalgae proteins had more than the RDI of the WHO/FAO (**Figure** **3**).

However, the biological values, digestibility, net protein utilization and protein efficiency ratio of microalgae proteins are reported to be lower than the gold standards, casein, and egg.^[^ ^7^ ^]^ This shortfall is because of the complex matrix of microalgae biomass where their proteins coexist and interact covalently and non‐covalently with several other biomolecules, thus making the proteins less digestible by gastrointestinal proteases and brush border peptidases. It is also known that the amount of proteins microalgae can accumulate is dependent on a number of factors, such as species type, growth phase and light quality.^[^ ^3^ ^]^ Given that these factors are amenable to modification through nutrient and environmental adjustments, the protein content and quality of microalgae may therefore be improved considerably by such modifications. More importantly, without prejudice to the nutritional potentials of microalgae, a pivot toward microalgae as sources of protein‐derived bioactive peptides for health promotion will free up conventional food proteins for nutritional purposes.

## 3 Processing Technology for Obtaining Peptides from Microalgae Proteins

**Figure 4:**
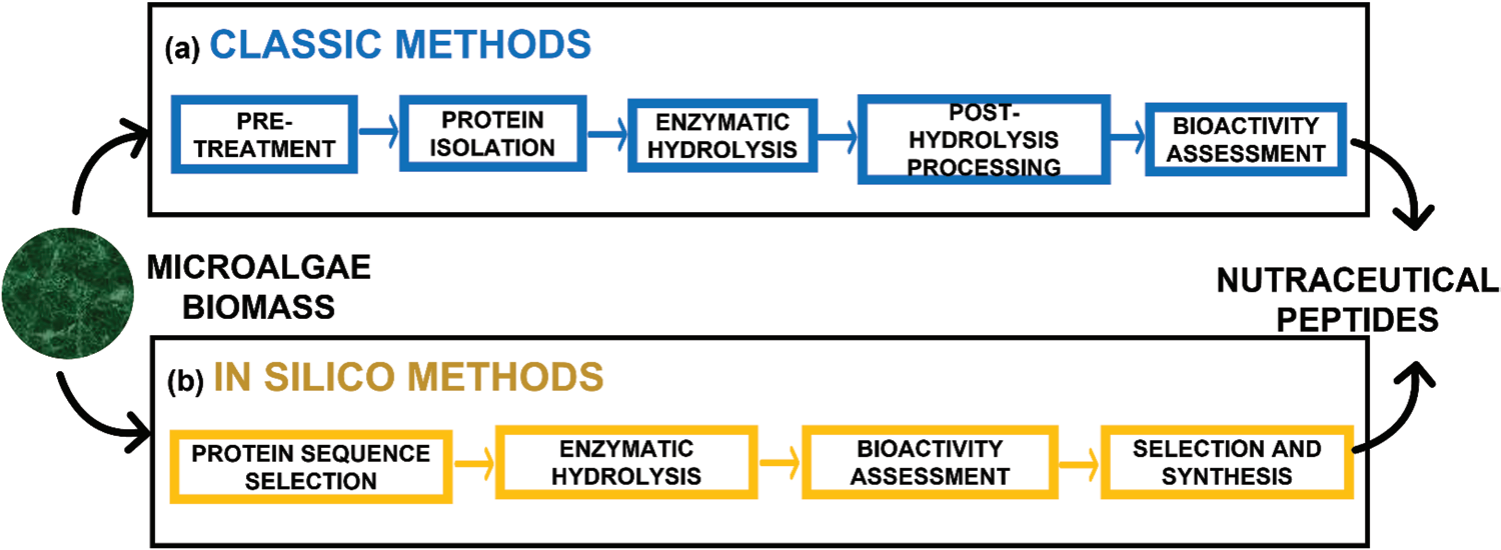
Classic/empirical and in silico methods used for the production and processing of nutraceutical peptides from microalgae proteins.

Production of bioactive peptides from microalgal proteins is usually affected by the recalcitrant nature of the cell walls of many microalgae. Microalgae cell walls usually have high‐viscosity and anionic cell‐wall polysaccharides and are known to be responsible for the reported poor digestibility, bioaccessibility, and bioavailability of their proteins. Mechanical, chemical, and enzymatic extraction methods are therefore used to breakdown the cell wall and release the proteins. The processes for generating peptides from microalgae are (for convenience) grouped into three stages—pre‐treatment and protein isolation, enzymatic hydrolysis of proteins, and post‐hydrolysis processing (**Table** **1** and **Figure** **4**)—and briefly discussed hereunder.

**Table 1:** Processing technologies used in the production of peptides

Group/classification	Specific technique	Specific example
Pre‐treatment and protein isolation	Mechanical techniques	Bead‐milling
		High‐pressure homogenization
		Ultrasonication
	Chemical treatment	Acidic digestion
		Alkaline digestion
		Enzymatic hydrolysis
	Non‐conventional treatments	Microwave‐assisted extraction
		Pulsed electric field extraction
		Subcritical and supercritical fluid extraction
		Steam explosion
		Hydrothermal liquefaction
Enzymatic hydrolysis of isolated proteins	In vivo by proteolysis with gastrointestinal enzymes	Simple proteolysis using pepsin, trypsin, α‐chymotrypsin, etc.
		Simulated gastrointestinal digestion (with pepsin for stomach phase digestion, and α‐chymotrypsin and trypsin, or pancreatin for intestinal digestion)
	In vitro by proteolysis with commercial enzymes	Protamex, Protease N, Alcalase, Nutrase, etc.
	In situ by proteolysis via microbial fermentation.	Protease‐producing microorganisms such as lactic acid bacteria
Post‐hydrolysis processing	Separation/fractionation of peptides	Centrifugation
		Precipitation
		Ultrafiltration
		Chromatography
		Electrophoresis
	Concentration	Freeze‐drying

### 3.1 Pre‐Treatments and Protein Isolation

Pre‐treatment enhances cell disruption and protein yield in microalgae.^[^ ^8^ ^]^ Mechanical techniques such as bead‐milling, high‐pressure homogenization and ultrasonication are typically used as pre‐treatment techniques prior to protein extraction; and have been shown to increase protein recovery from pre‐treated biomass by more than two‐folds compared to biomass that is not subjected to pre‐treatment.^[^ ^4^, ^9^ ^]^ Ultrasonication is based on the creation, multiplication, and collapse of bubbles formed by acoustic cavitation, which ultimately results in excitation and rupture of the cell wall.^[^ ^10^, ^11^ ^]^ Gerde et al.^[^ ^12^ ^]^ reported that ultrasonication (300 W, 20 kHz) at 50% amplitude and pH 11 for 4 min resulted in ≈60% higher protein yield from 1% solid suspension of non‐defatted *Nannochloropsis sp*. Ultrasonication pre‐treatment of 1% dry matter at 400 W for 30 min has also been shown to significantly improve the recovery of proteins from *Parachlorella kessleri* compared to untreated biomass, although high‐pressure homogenization produced higher extraction efficiency than ultrasound at the same energy level.^[^ ^13^ ^]^ Chemical extraction methods usually involve the use of aqueous, acidic, and/or alkaline media in digesting the microalgae biomass under varying conditions of temperature, pH, ionic strength, etc. Methods such as alkaline protein extraction by solubilization have been successfully used.^[^ ^14^ ^]^ Enzymatic disruption of microalgae cell walls (using enzymes such as xylanases, cellulases, lipase, proteases, lysozyme, amylases, autolysin, and pectinases) is widespread. The choice of enzyme is however dependent on chemical composition of the microalgae cell wall^[^ ^15^ ^]^ and the specific end use of the products as intact or pre‐hydrolyzed proteins. Chemical and enzymatic hydrolysis reactions are typically stopped by adjusting the pH or temperature.

Other (non‐conventional) means used in extracting microalgae proteins include microwave‐assisted extraction and pulsed electric field extraction. Microwave‐assisted extraction technique employs high‐frequency (300 MHz to 300 GHz) non‐ionizing electromagnetic radiation interacting with ionic and polar components of the microalgae to disrupt the cell walls and enhance the efficiency of protein extraction.^[^ ^16^, ^17^ ^]^ The pulsed electric field extraction technique uses high electric fields (100 to 300 kV cm^‐1^) to perforate cell membranes, thereby allowing the contents to leak into the medium.^[^ ^18^ ^]^ The use of subcritical and supercritical fluid extraction, steam explosion, and hydrothermal liquefaction techniques have also been reported.^[^ ^19^, ^20^ ^]^

### 3.2 Enzymatic Hydrolysis of Proteins

Enzymatic hydrolysis of proteins is the most described method for producing bioactive peptides.^[^ ^21^ ^]^ It is achieved in vivo by proteolysis with gastrointestinal enzymes, or in vitro by proteolysis with commercial enzymes, or in situ by proteolysis via microbial fermentation. Gastrointestinal enzymes, such as pepsin and trypsin, are commonly used in enzymatic hydrolysis. Other enzymes that are used include, Alcalase, α‐chymotrypsin, pancreatin, thermolysin, papain, subtilisin A, and other hydrolytic enzymes derived from bacterial or fungal sources. Enzymes exhibit extraordinary specificity, and this determines the type and quantity of peptides that result from the hydrolysis by specific enzymes. For example, trypsin hydrolysis is specific for peptide bonds on the C‐terminal side of lysine and arginine. Consequently, hydrolyzing a protein rich in lysine and arginine with trypsin will release many peptides, provided the lysine and arginine residues are not followed by a proline residue.^[^ ^22^ ^]^ Optimum conditions for enzymatic hydrolysis are usually pH 4–8 and temperature of 40—60 °C. This however is dependent on the known properties of the protease being used, and other relevant kinetic factors such as enzyme and substrate concentrations. In some cases, antioxidants are added to the protein broth before hydrolysis is initiated to ensure the redox stability of the protein hydrolysate. For the same reason, lipids and heme‐containing proteins are also removed prior to enzymatic hydrolysis.^[^ ^4^ ^]^ As mentioned earlier, at the end of hydrolysis, the reaction is stopped by adjusting the pH or temperature to inactivate the proteases.

Enzymatic hydrolysis has the advantage of being nontoxic and giving high yield of structurally diverse mixture of peptides. A combination of enzymes is also an option to obtain better results.^[^ ^23^, ^24^ ^]^ In proteolytic fermentation, microorganisms that produce proteases, such as lactic acid bacteria, are used to hydrolyze the protein substrate to release different polypeptides.^[^ ^25^ ^]^ The use of in situ microbial fermentation to release peptides however requires the optimization of the growth parameters of the microorganism in question. Indeed, different proteases can be used in a multistep recycling process coupled with an ultrafiltration membrane system to permit a series of enzymatic digestion, leading to the isolation of more bioactive peptides from the microalgae proteins.^[^ ^26^, ^27^ ^]^ Furthermore, simulated gastrointestinal digestion (wherein stomach phase digestion is simulated using pepsin while intestinal digestion is simulated using α‐chymotrypsin and trypsin, or pancreatin) is also a simple, efficient, and widely used model system.^[^ ^28^, ^29^, ^30^ ^]^

### 3.3 Post‐Hydrolysis Processing

Following hydrolysis (irrespective of the specific method used or how it is tweaked for efficiency), separation techniques such as centrifugation, precipitation, ultrafiltration, or chromatography, are deployed to separate the liberated peptides from the unhydrolyzed proteins.^[^ ^31^, ^32^ ^]^ Membrane ultrafiltration is commonly used in fractionating hydrolyzed microalgae protein into different molecular weight ranges.^[^ ^28^, ^30^, ^33^, ^34^ ^]^ This technique is typically used to achieve higher bioactivity by either concentrating the bioactive peptides of similar physicochemical properties (e.g., molecular size and net charge) into select fractions or the removal of inactive ones. Liquid chromatography has been effectively used for the separation of peptides.^[^ ^28^, ^35^ ^]^ For instance, Tejano et al.^[^ ^14^ ^]^ have used nano‐liquid chromatography‐nanoelectrospray ionization tandem mass spectrometry for microalgae peptide separation and identification. Other separation techniques (or modifications of major techniques) utilized, post‐hydrolysis, in the fractionation and purification of microalgae peptides, include size exclusion chromatography, affinity chromatography, and gel electrophoresis.^[^ ^29^, ^30^, ^36^ ^]^ After post‐hydrolysis processing, the resulting precipitate, filtrate, or supernatant, is usually concentrated by freeze‐drying to preserve the structural integrity of the peptides.

## 4 Biological Targets of Microalgae Peptides for Promoting Cardiovascular Health

Microalgae bioactive peptides have invaluable potential in fostering cardiovascular wellness and averting cardiovascular diseases.^[^ ^37^ ^]^ Some of these unique peptides from several microalgae species have high affinity and potential for interacting with targets to activate or inhibit their activities and physiological functions. They militate against the onset or progression of cardiovascular diseases and their enablers such as oxidative stress and inflammation.^[^ ^5^, ^38^ ^]^ In our previous review, older studies elucidating or reporting cardiovascular health‐promoting potentials of peptides from microalgae were thoroughly discussed, and prospects for future research were recommended.^[^ ^2^ ^]^ Here, we present an update on microalgae bioactive peptides with potentials for use in the management of hypertension, oxidative stress, dyslipidemia, and inflammation, published since our first review.

### 4.1 Hypertension

Hypertension has been recorded as one of the most common cardiovascular anomalies, characterized by a persistent increase in blood pressure beyond normalcy (high blood pressure). Hypertension fosters the onset of other co‐morbidities such as stroke, myocardial infarction, and heart failure.^[^ ^39^ ^]^ Although genetic factors and other risk factors like obesity may interplay to promote the onset of hypertension, several complex enzyme systems, and regulatory networks may be distorted during its progression.^[^ ^40^ ^]^ In recent studies, some of the regulatory enzyme networks have gained another function as targets for antihypertensive drugs, natural products, or bioactive peptides.^[^ ^38^ ^]^

#### 4.1.1 Angiotensin I‐Converting Enzyme as a Target for Hypertension

Among others, the renin‐angiotensin‐aldosterone system (RAAS) has been the most widely explored target in recent studies on the antihypertensive effects of microalgae‐derived bioactive peptides. Studies have shown that imbalances in the RAAS system pathway foster hypertension progression.^[^ ^41^ ^]^ Specifically, a drop in blood pressure triggers the cascade of enzymatic reactions in the RAAS system in a homeostatic state. First, the renin precursor protein (prorenin), which is abundant in juxtaglomerular cells of the kidney, is cleaved to renin and secreted into the blood. The renin enzyme acts on angiotensin (secreted from the liver), cleaving away a 10‐amino acid peptide to form inactive angiotensin I. Angiotensin I‐converting enzyme (ACE) (which holds a fulcrum position in the RAAS system, and is abundant in the vascular endothelia of the lungs and kidneys) converts angiotensin I to the potent vasoconstrictor, angiotensin II. The binding of angiotensin II to any of its receptors (such as angiotensin type IA, angiotensin type IB, angiotensin type II receptors) fosters vasoconstriction which increases blood pressure.^[^ ^42^ ^]^

Most recent studies on the bioactive peptides from microalgae showed their antihypertensive potential through their inhibitory activities and the downregulation of ACE expression. A recent investigation was conducted using combined in silico and in vitro approach to discover ACE‐inhibiting bioactive peptide from *A. platensis* strain C1.^[^ ^35^ ^]^ In the study, the computational tool, SpirPep, was used to predict that five optimal ACE‐inhibiting peptides fell within the phycobiliproteins, with the best peptide (labelled as SpirPep1) having the best half maximal inhibitory concentration (IC_50_) of 1.748 mm, as well as zero toxicity to fibroblasts cells of African green monkey kidney and human dermal skin. SpirPep1 showed the best binding energy with ACE protein (−883 kJ mol^−1^) from molecular docking and dynamics simulations.^[^ ^35^ ^]^ In another study, Montone et al.^[^ ^43^ ^]^ adopted a similar in silico analysis to discover four novel bioactive peptides (WPRGYFL, GPDRPKFLGPF, WYGPDRPKFL, SDWDRF) with excellent ACE inhibitory activities from proteins extracts of *Tetradesmus obliquus* microalgae, which was digested by alcalase and fractioned by 2D semi‐preparative reversed‐phase liquid chromatography. The in vitro ACE inhibitory studies gave a half maximal effective concentration (EC_50_) of 1060 and 7037 ng mL^−1^ for WYGPDRPKFL and GPDRPKFLGPF, respectively.^[^ ^43^ ^]^

Furthermore, Tejano et al.^[^ ^14^ ^]^ adopted in silico/bioinformatics approach to identify some peptides having ACE inhibitory activity from protein isolates of *Chlorella sorokiniana*. The microalgae gave a protein yield of 65.08%, of which eight different protein bands were clarified and identified by their SDS‐PAGE molecular weight, nanoLC–nanoESI MS/MS data, and alignment information from the NCBI databases. The BIOPEP‐UWM computational tools predicted the number of possible bioactive peptides with ACE inhibitory activities that exist within the different protein sequences, as well as the number of bioactive peptides that could be released from the different proteins when digested with different proteases. Amongst the identified proteins, the BIOPEP‐UWM tools predicted the following number of bioactive peptides with ACE inhibitory activities: 187 peptides from chloroplast Rubisco activase; 224 peptides from phosphoglycerate kinase; 276 peptides from heat shock protein 70; 204 peptides from ATP synthase β‐subunit (Chloroplast); 107 peptides from Fe‐superoxide dismutase; and 59 peptides from 50S ribosomal protein L7/L12 (chloroplast). Out of 15 different proteases explored with the BIOPEP‐UWM tools, three proteases, including pepsin, papain, and a combination of pepsin, trypsin, and chymotrypsin A, gave the highest number (31) of ACE‐inhibiting peptides. Despite the promising results, the predicted release of the microalgae bioactive peptides and molecular interactions with ACE need further validation using appropriate wet lab assays given the challenge of oversimplification often associated with in silico analysis.

Some studies have provided in vitro and in vivo evidence to support the ACE inhibitory activity of microalgae peptides. For instance, Lin and colleagues^[^ ^33^ ^]^ reported the isolation of four antihypertensive peptides from the byproduct of the hot water extraction (HWE) of a single‐cell green microalga (*C. sorokiniana)*. Protease N and gastrointestinal enzymes were used for the degradative transition of the HWE protein content to hydrolysate fractions and then to short peptide fragments. The most bioactive hydrolysate fraction (having a molecular weight between 270 and 340 Da) had the best in vitro inhibition of ACE with an IC_50_ of 0.015 mg mL^−1^. Moreover, four ACE‐inhibiting dipeptides (Trp‐Val, Val‐Trp, Ile‐Trp, and Leu‐Trp), isolated and purified from the most‐active fraction, showed IC_50_ values of 307.61, 0.58, 0.50, and 1.11 µm, respectively.^[^ ^33^ ^]^ Finally, in vivo experiments on hypertensive rats fed with the protease N hydrolysate powder at a dosage of 171.4 mg kg^−1^ body weight caused a reduction in the systolic and diastolic blood pressure by 20 and 21 mm Hg, respectively, after 6 h of oral administration.^[^ ^33^ ^]^ Therefore, *C. sorokiniana* proteins, obtained from hot water extraction, holds great promise as a sustainable source of potent bioactive peptides for reducing hypertension and improving cardiovascular health.

In another study, a species from the genus Chlorella (*C. vulgaris*) was screened for ACE‐inhibiting peptides. After in silico gastrointestinal digestion of the extracted protein, two novel tripeptides (Thr‐Thr‐Trp and Val‐His‐Trp) with IC_50_ of 0.61 and 0.91 µm, respectively, were discovered. Through molecular docking analysis, a strong interaction (6 hydrogen bonds) was found between the peptides and the amino acid residues of the active site of ACE.^[^ ^44^ ^]^ Additionally, the in vivo administration of the tripeptides to spontaneously hypertensive rats at a dosage of 5 mg kg^−1^ body weight caused a significant reduction in systolic blood pressure. A cumulative reduction of the systolic blood pressure by 31 mmHg was achieved at the end of the experiment with Val‐His‐Trp, whereas the positive control (treatment with the same dose of Lisinopril) only caused a 10‐mmHg drop. Both peptides Thr‐Thr‐Trp and Val‐His‐Trp caused a decrease in the diastolic blood pressure from 180–140 mmHg at 2 h and 174–153 mmHg at 1 h, respectively.^[^ ^44^ ^]^ Other studies have reported novel bioactive peptides from other microalgae species (*Gracilariopsis lemaneiformis, Chlamydomonas reinhardtii*, and others) with inhibitory activities against ACE.^[^ ^28^, ^45^ ^]^ **Table** **2** summarizes the relevant details for bioactive peptides with antihypertensive or blood pressure lowering properties published in the period under review.

**Table 2:** Bioactive peptides derived from microalgae proteins with reported antihypertensive and lipid modulating properties

S/N	Name of microalgae	Amino acid/bioactive peptide sequence	Specific target enzyme/ system	Type of study/model used	IC_50_ / EC_50_ or tested dose	References
1	Chlorella sorokiniana	a) NFNNIEDGFYISPAFLDK (in vitro) b) LVDAFPGQSIDFFGALR (in vitro) c) potentially 224 ACE inhibitor peptides	–	In silico	–	Tejano et al.^[^ ^14^ ^]^
2	Arthrospira platensis C1	IRDLDYY	–	In silico and in vitro	1.748 mm	Anekthanakul et al.^[^ ^35^ ^]^
3	*Tetradesmus obliquus*	a) GPDRPKFLGPF b) WYGPDRPKFL	–	In vitro	5.73 µmol L^−1^ (a) 0.82 µmol L^−1^(b)	Montone et al.^[^ ^43^ ^]^
4	*Isochrysis zhanjiangensis*	Phe‐Glu‐Ile‐His‐Cys‐Cys (FEIHCC)	NF‐κB DNA binding, MAPK, Nrf2, Akt pathways	In vitro and ex vivo (HUVEC)	10‐100 µM.	Chen et al.^[^ ^52^ ^]^
5	*Spirulina platensis*	a) NSLGVPI, b) GIVAGDVTPI	PI3K/AKT/eNOS pathway	Ex vivo (rat mesenteric arteries), in vivo (eNOS‐knockout mice)	200 µg mL^−1^ (ex vivo) 10 mg kg^−1^ (in vivo)	Carrizzo et al.^[^ ^51^ ^]^
6	Chlorella sorokiniana	a) Trp‐Val b) Val‐Trp c) Ile‐Trp d) Leu‐Trp	Angiotensin converting enzyme	In vitro and in vivo (SHR)	307.61, 0.58, 0.50, and 1.11 µm respectively(in vitro); 85.7 and 171.4 mg kg^−1^ of hydrolysate (in vivo)	Lin et al.^[^ ^33^ ^]^
7	*Chlorella vulgaris*	a) Thr‐Thr‐Trp (TTW) b) Val‐His‐Trp (VHW)	Angiotensin converting enzyme	In vitro and in vivo (SHR)	0.61 ± 0.12 µm (TTW) and 0.91 ± 0.31 µm (VHW)	Xie et al.^[^ ^44^ ^]^
8	*Gracilariopsis lemaneiformis*	a) FQINM(O)CILR b) TGAPCR	Angiotensin converting enzyme	In vitro and in vivo (SHR)	9.64 and 23.94 µm (in vitro); 10 mg mL^−1^ in vivo	Deng et al.^[^ ^28^ ^]^
9	*Spirulina platensis*	Hydrolysate composed of 217 peptide sequences	–	In vivo (high‐fat diet fed rats)	150 mg kg^−1^ per day	Hua et al.^[^ ^53^ ^]^
10	*Chlamydomonas reinhardtii*	RPLKPW, LKPNM, and AINPSK (cloned into a fusion protein) RPLKPW and AINPSK (detected after enzymatic hydrolysis of recombinant protein)	Peptides have known ACE inhibition, nitric oxide‐dependent vasorelaxation properties.	In vivo (SHR)	10 mg kg^−1^ of recombinant protein	Carrizalez‐López et al.^[^ ^45^ ^]^

#### 4.1.2 Endothelial Nitric Oxide Synthase (eNOS) as a Target for Protection Against Hypertension

The eNOS is one interesting pro‐target for protection against hypertension and cardiovascular diseases, as it produces nitric oxide (NO), which protects vascular integrity by preventing platelet aggregation, and smooth muscle proliferation.^[^ ^46^ ^]^ NO is usually produced from the oxidation of the l‐arginine to l‐citrulline by eNOS in the presence of the 5,6,7,8‐tetrahydro‐l‐biopterin (BH_4_) cofactor. Oxidative stress, such as the abundance of superoxide radical in the cells, mops up the vasoprotective NO to produce peroxynitrite (ONOO^−^), which then diminishes the BH_4_ cofactors. The reduction in the level of the BH_4_ cofactors causes the uncoupling of eNOS from its enzyme complex; and fosters the production of more superoxide radical.^[^ ^47^ ^]^ Moreover, phosphorylation of eNOS has been shown to promote the NO production activities of eNOS and hence cardiac protection.^[^ ^48^ ^]^ Several signaling paths foster the activation of eNOS for NO production. The phosphoinositide‐3‐kinase/serine/threonine kinase Akt (PI3K/AKT) pathway is one focus of contemporary research.^[^ ^49^ ^]^ In this signaling cascade, the PI3Ks, which are usually activated by G‐protein coupled receptors and receptor tyrosine kinases, phosphorylate, and activate the serine/threonine kinase Akt, which in turn activates eNOS for NO production and vasoprotection. Hence, cardio‐protective bioactive compounds may target this signaling cascade to promote NO generation and vasodilation.^[^ ^50^ ^]^

Recent studies have continued to report microalgae bioactive peptides with blood pressure‐reducing potentials through PI3K/AKT/eNOS signaling cascades. For example, Carrizzo and colleagues^[^ ^51^ ^]^ isolated a novel decameric peptide from microalga *Spirulina platensis* with the potential to increase the vascular generation of NO as a vasoprotectant in resistance vessels of mice.^[^ ^51^ ^]^ In their study, *S. platensis* protein fraction was produced using an in vitro simulation of the gastrointestinal digestive process. The crude fraction with the best vasorelaxation potential was analyzed by high‐resolution mass spectrometry, and the most potent peptide (GIVAGDVTPI) was identified.^[^ ^51^ ^]^ The novel decapeptide induced vasodilation and hemodynamic effects by stimulating the PI3K/AKT/eNOS axis. The vasorelaxation and hemodynamic effects were endothelium‐dependent, and no effects were observed in eNOS knockout mice.^[^ ^51^ ^]^ This study has exposed the PI3K/AKT/eNOS axis as another option for future studies investigating the cardioprotective effects of microalgae‐derived bioactive peptides other than the RAS targets.

### 4.2 Dyslipidemia

Dyslipidemia, one of the risk factors of cardiovascular diseases, involves the imbalances in different lipid classes such as cholesterol, low‐density lipoprotein cholesterol (LDL‐C), high‐density lipoprotein cholesterol (HDL‐C), and triacylglycerols.^[^ ^54^ ^]^ Generally, improving serum HDL‐C concentrations prevents cardiovascular diseases, while an increase in LDL‐C promotes cardiovascular diseases.^[^ ^55^ ^]^ Therefore, these lipoprotein‐containing cholesterol fractions are potent targets for combating cardiovascular diseases and promoting cardiovascular health. Bioactive compounds targeting enzymes or cofactors that activate or deactivate the lipid cholesterol complexes and maintain their homeostasis could be used to promote cardiovascular health.^[^ ^56^ ^]^

Bioactive peptides from microalgae have been shown in recent studies to act in ways to prevent cardiovascular diseases (Table 2). Hua et al.^[^ ^53^ ^]^ used the protease Protamex to hydrolyze *S. platensis* and investigated the serum lipid modulating properties of the product in rats. They reported that the *S. platensis* protease hydrolysate composed of 217 peptide sequences; and that when given to high‐fat diet rats at a dose of 150 mg kg^−1^ bodyweight for four weeks, the treatment resulted in significant decreases in serum total cholesterol, triacylglycerols and low‐density lipoprotein cholesterol concentrations of the rats. The observed patterns were present even after 8 weeks, and the high‐density lipoprotein‐cholesterol level was significantly higher in the test group at 8 weeks compared to the control. The authors concluded that “*Spirulina platensis* peptides have the potential to ameliorate lipid metabolic disorders”. The peptides therefore could play significant roles in the prevention and management of cardiovascular diseases.

### 4.3 Oxidative Stress

Oxidative stress is one critical metabolic factor that fosters cardiovascular disease onset and progression. Oxidative stress plays a central role in cardiovascular pathology, as it is implicated in atherosclerotic risk factors—hypertension, dyslipidemia, inflammation, and obesity—which are components of the metabolic syndrome.^[^ ^57^ ^]^ If not managed properly, the reactive oxygen species (ROS) generated from different metabolic activities could overwhelm the inherent activities of cellular antioxidant systems, and result in several disorders, including cardiovascular diseases.^[^ ^58^ ^]^ Using antioxidant chemicals or products from natural sources like microalgae is an alternative means of mopping up ROS from biological systems.^[^ ^59^ ^]^

Bioactive peptides from microalgae have been widely reported for their antioxidant capacity. Some of these peptides can scavenge free radicals and reactive species, thereby reducing oxidative stress. Alternatively, the bioactive peptides may act as effectors of antioxidant enzymes, thereby boosting their activities or even upregulating relevant gene expression.^[^ ^60^ ^]^ Several recent studies have demonstrated the anti‐oxidative effects of bioactive peptides from microalgae to include both radical scavenging activities and promotion of the activities of antioxidant enzymes. Moreover, other studies have predicted the antioxidant activities of microalgae bioactive peptides using in silico and computational tools such as BIOPEP‐UWM. In a study using the BIOPEP‐UWM tool (with pepsin [EC 3.4.23.1] as hydrolytic enzyme), Tejano et al.^[^ ^14^ ^]^ predicted the abundance of 11 antioxidant bioactive peptides inherent within the heat shock protein of *C. sorokiniana*. The study also identified VPL, WG, LA, IR, PG, VY, and KP as the bioactive peptides in *C. sorokiniana* with multiple activities, including antioxidant and ACE inhibitory activities.^[^ ^14^ ^]^

In another study, the microalgae biomass of Arthrospira maxima obtained from the bioethanol production chain using sugarcane vinasse was shown to have an abundance of bioactive peptides possessing antioxidant, anti‐inflammatory, and other bioactivities.^[^ ^61^ ^]^ The protein content of the biomass was extracted by four freeze‐thaw cycles, homogenization, and ultra‐sonication. From the protein, three hydrolysates were prepared using two endopeptidases—subtilisin A and pepsin. The first hydrolysate was prepared with subtilisin A (PHA), the second with pepsin (PHP), and the third with both endopeptidases (PHS). The antioxidant capacity of the three hydrolysates was shown via several in vitro studies. At a concentration of 0.1 g mL^−1^, the three hydrolysates showed 77–78% scavenging of the free DPPH radical, and their IC_50_ ranged from 17.93 to 34.63 µg mL^−1^. The PHA and PHS hydrolysates were reported with IC_50_ values for ABTS^+^ radical scavenging of 9.5 and 8.6 µg mL^−1^, and a TEAC value of 465.7 and 540.7 Trolox µm g^−1^, respectively. Finally, the PHP peptide fraction was reported to have the best iron‐chelating activity with about 97.3% iron chelated at a concentration of 25 µg mL^−1^ and IC_50_ of 0.007 mg mL^−1^. The unique iron‐chelating activities of the PHP peptides suggest their potential to trap the circulating non‐heme iron that often cause an increase in the cell redox potential and oxidative stress.^[^ ^61^ ^]^

Furthermore, Xia et al.^[^ ^36^ ^]^ hydrolyzed *Dunaliella salina* protein through in vitro gastrointestinal digestion and membrane ultrafiltration. The study adopted the DPPH^+^ scavenging assay to investigate the antioxidant activities of the peptides from the protein. It was shown that protein digestion with proteases caused an improvement in the antioxidant capacity. The total digested peptide gave 85% DPPH scavenging activities which was about 1.5 times higher than the undigested sample. Moreover, the protein fraction with molecular weight between 500–1000 Da showed the highest DPPH scavenging activities (81.2%) with the abundance of four novel peptides (ILTKAAIEGK, IIYFQGK, NDPSTVK, and TVRPPQR) detected by RPLC‐Q HF mass spectrometry.^[^ ^36^ ^]^ In a different study, however, a higher molecular weight peptide fraction (5000–10 000 Da) from *Schizochytrium limacinum* protein was reported to have the best antioxidant and scavenging activities for DPPH and ABTS radicals.^[^ ^30^ ^]^ It is noteworthy that Xia et al.^[^ ^36^ ^]^ reported that novel peptides (ILTKAAIEGK, IIYFQGK, NDPSTVK, and TVRPPQR) had an abundance of hydrophobic amino acids (about 56%), which probably accounts for their remarkable antioxidant properties. Indeed, Zhang et al.^[^ ^62^ ^]^ reported that a high ratio of hydrophobic amino acids relative to hydrophilic ones, and the smaller molecular size of a peptide, improve its antioxidant capacity. In their study, the peptide Glu‐Leu‐Trp‐Lys‐Thr‐Phe was isolated from the chymotrypsin hydrolysate of *Gracilariopsis lemaneiformis* proteins and was found with a DPPH scavenging activity (EC_50_, 1.514 mg mL^−1^).^[^ ^62^ ^]^ The studies which reported the antioxidant potencies of many peptides from different microalgae using in vitro experiments are summarized in **Table** **3**.

**Table 3:** Bioactive peptides derived from microalgae proteins with reported antioxidant activity

S/N	Name of microalgae	Amino acid/bioactive peptide sequence	Specific target enzyme/ system	Type of study/model used	IC_50_ / EC_50_ or tested dose	References
1	Chlorella sorokiniana	a) NFNNIEDGFYISPAFLDK (in vitro) b) LVDAFPGQSIDFFGALR (in vitro) c) potentially 224 antioxidant peptides	–	In silico	–	Tejano et al.^[^ ^14^ ^]^
2	Arthrospira maxima OF15	20 peptides (subtilisin A = 4, pepsin = 15, both = 1); unreported sequences	–	In vitro	21.3; 34.6 and 17.9 µg mL^−1^, respectively	Montalvo et al.^[^ ^61^ ^]^
3	*Chlorella protothecoides*	Protein hydrolysates of molecular weight <46 kDa	–	In vitro	Not reported	Olena et al.^[^ ^24^ ^]^
4	*Dunaliella salina*	(a) Ile‐Leu‐Thr‐Lys‐Ala‐Ala‐Ile‐Glu‐Gly‐Lys (b) Ile‐Ile‐Tyr‐Phe‐Gln‐Gly‐Lys (c) Asn‐Asp‐Pro‐Ser‐Thr‐Val‐Lys (d) Thr‐Val‐Arg‐Pro‐Pro‐Gln‐Arg	–	In vitro	100 mL	Xia et al.^[^ ^36^ ^]^
5	*Gracilariopsis lemaneiformis*	ELWKTF	–	In vitro	1.514 mg mL^−1^	Zhang et al.^[^ ^62^ ^]^
6	*Schizochytrium limacinum*	Uncharacterized 5–10 kDa peptide fraction	–	In vitro	100–1000 µL of sample	Moaveni et al.^[^ ^30^ ^]^
7	*Tetradesmus obliquus*	a) WPRGYFL b) SDWDRF	–	In vitro	4.70 µmol L^−1^ (ABTS) for (a); 13.97 µmol L^−1^ (DPPH) for (b)	Montone et al.^[^ ^43^ ^]^
8	*Arthrospira platensis*	VKYVSPTCGPCH	Free radicals scavenging	In vitro, ex vivo and in vivo	6.25; 12.5;20 µm, respectively	Sannasimuthu and Arockiaraj^[^ ^63^ ^]^
9	*Arthrospira platensis*	GGGAFSGKDPTKVDR	Up‐regulation of glutathione family gene expression	In vitro, ex vivo and in vivo	5‐45 µm	Velayutham et al.^[^ ^64^ ^]^
10	*Isochrysis zhanjiangensis*	NDAEYGICGF	Regulation of SOD, GSH, and GGT expression	In vitro and ex vivo	25‐50 µL (in vivo) 10–100 µM (ex vivo)	Chen et al.^[^ ^29^ ^]^
11	*Synechococcus sp* VDW	a) AILQSYSAGKTK b) ALNKTHLIQTK c) LLVHAPVK d) IPDAHPVK e) VVVLRDGAVQQLGTPR	Reduced pro‐inflammatory gene expression	In vitro and ex vivo	In vitro: 34.51 µg mL^−1^ (MW < 3 kDa fraction); Ex vivo: 3.75 to 120 µg mL^−1^ (MW < 3 kDa fraction)	Suttisuwan et al.^[^ ^22^ ^]^
12	*Isochrysis zhanjiangensis*	EMFGTSSET	MAPK, NF‐κB) and Akt signal pathways	Ex vivo	10–100 µm	Pei et al.^[^ ^67^ ^]^

Other experimental approaches (ex vivo and in vivo) have been adopted by recent studies to demonstrate the anti‐oxidative potentials of peptides from microalgae species (Table 3). Using in vitro, ex vivo, and in vivo experimental approaches, Sannasimuthu and Arockiaraj^[^ ^63^ ^]^ reported the impressive antioxidant activities of a peptide (VH12) derived from the thioredoxin disulfide reductase protein of *A. platensis*. From the in vitro studies, it was seen that VH12 scavenged DPPH radical, eliminating more than 50.0% and 98.5% of the free radicals at low (6.25 µm) and high (200 µm) concentrations, respectively. The *ex vivo* experiments showed that the ROS level in cardiac myocyte cells was reduced by 55% when treated with 12.5 µm of VH12 peptides. Moreover, the peptide preparation showed no cytotoxic effects on human leucocyte cells. Finally, the in vivo experiment with zebrafish embryo showed that the VH12 peptide at 20 µm significantly reduced the H_2_O_2_‐induced oxidative stress, and this was demonstrated by the low‐level fluorescence [tested using 2′,7′‐dichlorofluorescein diacetate (DCFDA) dye] emitted from treated embryo challenged with 500 µm H_2_O_2_.^[^ ^63^ ^]^

Velayutham and colleagues^[^ ^64^ ^]^ reported the antioxidant properties of a novel peptide, GR15, constituting majorly aliphatic amino acids (GGGAFSGKDPTKVDR) from S‐adenosylmethionine synthase (SAMe) protein of *A. platensis* through in silico, ex vivo and in vivo experimental methodology. In the in silico analysis, the physiochemical properties of the SAMe protein and GR15 peptide were predicted. It was also reported that the mRNA of SAMe in *A. platensis* was upregulated by 3–6‐folds when grown under oxidative stress conditions for 5 to 15 days. Moreover, the in vitro assay showed that GR15 at a concentration of 45 µm reduced the free ABTS radicals by 82.56%, superoxide radicals by 73.45%, hydrogen peroxide by 78.45 and nitric oxide radicals by 77.12%. Also, GR15 was non‐cytotoxic to MDCK cells as LDH releases were insignificant for all the treatment doses (5‐45 µm), and the percentage of cell viability was above 75% in all treatment groups. *Ex vivo* antioxidant activities of GR15 were assayed by monitoring the superoxide dismutase (SOD) and catalase (CAT) activities in oxidatively stressed MDCK cells treated with 5–45 µm GR15. The MDCK cells treated with 45 µm GR15 showed SOD activities of 0.392 units per 10 µL and CAT activities of 0.676 units per 10 µL. In contrast, the control group (no oxidative stress) had 0.485 units per 10 µL and 0.735 units per 10 µL activities for SOD and CAT, respectively. The remarkable boost of antioxidant activities by GR15 in the MDCK cells was demonstrated by the comparison with the enzyme activities of the untreated control challenged with 200 µm H_2_O_2_ (0.056 units per 10 µL and 0.095 units per 10 µL for SOD and, CAT respectively).^[^ ^64^ ^]^ Similarly, in the zebrafish embryo, 45 µm of GR15 caused improvement of SOD and CAT enzyme activities from less than 10 to 30 and 27 U mg^−1^ protein, respectively. Also, the malondialdehyde (MDA) level showed a dose‐dependent decrease (from 60 to 15 mMol min^−1^ mg^−1^ protein) on treatment with GR15. More so, there was an upregulation in mRNA expression of glutathione peroxidase (GPx), glutamyl cysteine synthetase (GCS), and glutathione S‐transferase (GST) genes by 11.5‐, 15‐, and 10.37‐folds, respectively in treated cells. Finally, GR15 promoted the reduction of oxidative stress on MDCK cells caused by 200 µm H_2_O_2_ in a dose‐dependent fashion, from 79.97% to 33.59%.^[^ ^64^ ^]^

### 4.4 Inflammation

Inflammation is a two‐edged sword, with beneficial and harmful effects. Inflammation is beneficial as it occurs due to the recruitment and action of immune cells around an infection or foreign body. However, the prolonged effect or overproduction of these immune cells causes systemic disorders characterized by pain, redness, and swelling.^[^ ^65^ ^]^ Inflammation of the endothelial cells causes the narrowing of the blood vessels and hence increased blood pressure. Moreover, some pro‐inflammatory mediators could clog and form plaque on the walls of blood vessels, blocking them and causing ischemic and heart attacks.^[^ ^66^ ^]^ Therefore, receptors and immune cells that foster the production of these pro‐inflammatory molecules are potential targets for cardiovascular drugs and natural products with cardioprotective effects such as microalgae bioactive peptides.

In the study by Montalvo et al.,^[^ ^61^ ^]^ the anti‐inflammatory activities of bioactive hydrolysate fractions from *A. maxima* OF15 biomass (PHA, PHP, and PHS as previously described) were evaluated by the in vitro hyaluronidase (HA) (Type IV) inhibition assay. The enzyme hyaluronidase has been reported to recruit pro‐inflammatory markers. Its inhibition by the peptides showed their potencies to prevent inflammation via this route. The three peptide fractions all exhibited anti‐hyaluronidase activities in dose‐dependent fashions. Moreover, the PHS peptide had the best activities, causing a 38.8% inhibition of HA at 333 µg mL^−1^and the lowest IC_50_ at 0.92 mg mL^−1^. The other peptide fractions, PHA and PHP, gave an IC_50_ of 1.63 and 1.66 mg mL^−1^, respectively.^[^ ^61^ ^]^

In another study, bioactive peptides were prepared from the microalgae, *Synechococcus* *sp* by the trypsin digestion of its proteins and ultrafiltration with a molecular weight cut‐off membrane for 3, 5, and 10 kDa sizes.^[^ ^22^ ^]^ The peptide fractions with MW of 3 kDa and below showed no cytotoxicity to RAW264.7 macrophage cells. It promoted the downregulation of pro‐inflammatory cytokines such as iNOS, TNF‐α, COX‐2, and IL‐6. On further analysis of the fraction (MW <3 kDa) with reverse phase HPLC techniques and Q‐TOF ESI mass spectrometry, five bioactive peptides were identified, including AILQSYSAGKTK, ALNKTHLIQTK, LLVHAPVK, IPDAHPVK, and VVVLRDGAVQQLGTPR, all contributing to the excellent anti‐inflammatory properties of the protein fraction.^[^ ^22^ ^]^

Finally, a novel nonapeptide (EMFGTSSET) isolated from *Isochrysis zhanjiangensis* was reported with potent anti‐inflammatory as well as anti‐apoptotic activities against oxidized LDL‐induced toxicity and atherosclerosis in HUVEC cells.^[^ ^67^ ^]^ The nonameric peptide was isolated by in vitro simulated gastrointestinal digestion of the *I. zhanjiangensis* protein extract, which fostered the upregulation of anti‐inflammatory and anti‐apoptotic markers while repressing the expression of pro‐inflammation molecules. In detail, the nonapeptide inhibited the expression of oxidized LDL receptor 1 (LOX‐1) induced by the oxidized LDL and reduced oxidative stress caused by ROS on cells. Moreover, the peptide inhibited the production of pro‐inflammatory molecules (such as IL‐6, IL‐1β, and TNF‐α) and cell adhesion molecules (VCAM 1 and ICAM 1). More so, the expression of p‐p65, p‐IκB‐α, p‐p38, p‐ERK, p‐JNK, bax, and cleaved caspase‐9/‐3, which are necessarily expressed during the oxidized‐LDL‐induced inflammation and atherosclerosis, were down‐regulated. Whereas anti‐inflammatory molecules [such as nuclear red blood cell 2 related factors 2 (Nrf2), heme oxygenase‐1 (HO ‐1), p‐Akt, and bcl‐2] were up‐regulated. The anti‐inflammatory activities of the peptide were supported by findings from molecular docking and in silico studies, as a strong binding interaction of the nonapeptide to LOX‐1 and VCAM‐1 was reported.^[^ ^67^ ^]^

## 5 Challenges to Practical Application of Microalgae Peptides

Although microalgae are promising sources of bioactive peptides (owing to their diverse protein reservoir) that are useful in the management of cardiovascular diseases and other health conditions, a number of challenges militate against their widespread utilization. The challenges are typically with respect to biomass production, downstream processing, safety, and the inherent characteristics of the eventual bioactive peptides.

The economics of microalgae production does not yet favor the commercialization of microalgae‐based commodities. Only algal species such as *Chlorella*, *Spirulina*, *Nannochloropsis*, *Isochrysis*, *Porphyridium*, and *Dunaliella* have shown commercial potential; yet, even for these few, large‐scale production is limited by their slow growth rate and its attendant risk of contamination.^[^ ^68^ ^]^ Microalgae species that are amenable to large‐scale cultivation and which grow rapidly are needed. Alternatively, research into process optimization (by tweaking biotic and abiotic factors) could lead to enhanced biomass production even in promising species. The fact that most microalgae are extremophiles, capable of surviving and thriving in a wide array of environmental conditions, make process optimization plausible.

Harvesting and downstream processing of microalgal biomass are very challenging processes. Since the production of bioactive peptides requires large amounts of biomass, and continuous operational centrifugation is superior to flocculation when large amounts of biomass are needed, the use of self‐flocculating algal species like *C. vulgaris* JSC7, which may lower costs, is inadequate. Species that yield biomass with high solid content and are amenable to efficient drying methods (such as sun drying, air drying, vacuum drying, and spray drying) could make up for the economic costs of harvesting especially in the tropics.^[^ ^69^ ^]^ Yet, traditional processing techniques that include processes such as heating may impart the peptides negatively. Heating is known to affect the chemistry of proteins and often affect the activity and availability of the peptides. In addition to freeze‐drying, some new technologies, such as infrared radiation drying and microwave combined drying are available and appear to spare proteins more than traditional drying techniques.^[^ ^70^ ^]^

Furthermore, chemical and physical processing methods and storage conditions may affect peptides bioactivities and bioavailability due to the inherent chemical or physical changes they cause.^[^ ^71^, ^72^ ^]^ Additionally, the polysaccharide content of the microalgae cell wall hinders protein extraction as it affects protein yield, purity, and digestibility.^[^ ^73^ ^]^ Processing may also lead to the production of undesirable compounds such as d‐amino acids and biogenic amines. It is thought that peptide/food matrix interactions resulting from processing may negatively affect the bioactivities of peptides.^[^ ^71^, ^74^ ^]^ It is therefore important to research and develop more cost‐effective and efficient methods that guarantee high yield of peptides from their parent microalgal protein sources while sparing their native structure and biological activity.

Some microalgae may have proteins capable of triggering an immune response by the immunoglobulin E (IgE) antibody and are therefore classified as allergens. The immune response may be in the form of rashes, eczema, rhinitis, fever, etc. The enzymatic hydrolysis of the proteins to yield peptides, especially using proteases of limited specificity, may not significantly alter the allergenicity of the parent protein. Therefore, bioactive peptides from an allergenic protein are likely to remain allergens.^[^ ^75^ ^]^ Furthermore, non‐digestible proteins are thought to contribute to increased risk of some chronic diseases such as cardiovascular diseases.^[^ ^76^ ^]^ Therefore, in vivo studies are needed to exclude allergenicity, ensure tolerability and safety, of microalgal peptides with potentials in health promotion.

Protein hydrolysates are associated with a bitter taste, which could be a problem for substances taken orally.^[^ ^77^, ^78^ ^]^ Processes such as enzymatic debittering using exopeptidases, Maillard reaction, plastein reaction, and (micro)encapsulation have been developed to combat the said undesirable flavor.^[^ ^78^ ^]^ For example, it is reported that using octenyl succinic anhydride (OSA) starch to coat peptides can conceal the bitter taste of microalgae peptides. These techniques often involve the addition of new non‐bioactive materials into the nutraceutical product matrix. To reduce excessive processing and cost of production, more research is required to generate microalgae bioactive peptides with desirable taste profiles or to improve effectiveness and efficiency of the techniques used in overcoming the bitter taste of the peptides without compromising quality, potency, or affordability.

Bioactive peptides are also amenable to digestion by gastrointestinal enzymes. Implicit in this is the possibility that the bioactive peptide may be hydrolyzed by specific enzymes and therefore lose its activity. Again, akin to the interactions between the bioactive peptides and the matrix of the delivery vehicle, peptide interactions with the food contents of the gastrointestinal tract can also affect their bioaccessibility, bioavailability, or bioactivity. Studies have shown that synthesized bioactive peptides when subjected to simulated gastrointestinal digestion have attenuated or accentuated activities,^[^ ^79^ ^]^ and that the length or size, amino acid composition, and physicochemical properties of a peptide are central to whether it is modified by digestive enzymes. Testing for the stability of identified bioactive peptides in the presence of gastrointestinal enzymes is therefore central to the development of microalgae peptides for use in managing cardiovascular diseases.

Finally, given than most peptides derived from microalgae have been tested largely in vitro,^[^ ^23^ ^]^ more in vivo studies are needed to validate the claims from in silico, in vitro, and ex vivo studies on the promise of peptides from microalgae in cardiovascular disease management. Filling this knowledge gap will help to establish clear evidence of the competitiveness of microalgae as a sustainable source of nutraceutical bioactive peptides compared to the other alternative protein sources.

## 6 Conclusion and Future Directions

Microalgae continue to hold strong promise as sustainable raw materials for industry‐scale production of bioactive peptides for combating cardiovascular diseases. This is because of the relative expeditiousness of production of high amounts of protein‐rich microalgae biomass, and the high nutritional quality of microalgae proteins compared to plant‐ and animal‐derived proteins. In this review, we have provided recently published evidence on the processing technology used to generate bioactive peptides from microalgae proteins as well as their various cardiovascular‐related bioactivities. Also provided in this paper are several considerations that impede the full realization of these potentials. For instance, it is impractical to upscale some innovations, such as the purified microalgae bioactive peptides discovered in some studies, because of high cost of production and extremely low yields of the products, even with abundant microalgal biomass. Considering all the issues presented, we propose that future R&D investments should focus on the following specific areas to move microalgae‐derived peptides from bench to consumers:

1) Protein isolation and bioactive peptide production and processing scale‐up.
2) New product development with peptide‐rich bioactive hydrolysates for mainstream and niche markets.
3) Efficacy testing in susceptible populations for cardiovascular benefits.
4) Safety evaluation of hydrolysates and formulated products, especially for allergenicity.
5) Consumer acceptability evaluation for the novel functional ingredients.

Simultaneously, mechanistic studies should be conducted to understand the detailed array and uniqueness of the beneficial effects caused by microalgae peptides relative to bioactive peptides from other sources. This point is relevant because microalgae can produce a diverse profile of proteins, with wide sequence variability, dependent of their growth conditions. This provides a tremendous resource for generating massive combinations of peptide pharmacophores with a wide range of molecular targets and potentially novel mechanisms of action. These future directions will enhance understanding of the structure and biofunctional properties of microalgae proteins and peptides, and their practical utilization in nutraceuticals and functional foods to provide nutritional and cardiovascular health benefits to consumers—big things in small packages.

## Conflict of Interest

The authors declare no conflict of interest.

## Author Contributions

Conception: CCU; Design: C.C.U., C.E.C.C.E.; Literature search and data mining: C.C.U., C.E.C.C.E., O.A., T.C.P.E.; Manuscript writing: C.E.C.C.E., T.P.C.E., C.C.U., O.A.; Manuscript vetting and revision: C.E.C.C.E., C.C.U.; Approval for publication: C.C.U., C.E.C.C.E., T.P.C.E., O.A.
